# Data on self-medication among healthcare students at Najran University, KSA

**DOI:** 10.6026/97320630017599

**Published:** 2021-05-31

**Authors:** Siraj DAA Khan, Musleh Al-Garni, Faisal Ali Alalhareth, Abdulellah Abdulslam Al Touk, Hamoud Abdullah Al-Ajmi, Saeed Ali Alyami, Hamzah Hamed Alalyani

**Affiliations:** 1 Department of Preventive Dental Sciences, Faculty of Dentistry, Najran University, KSA

## Abstract

The prevalence of self-medication (SM) has increased in health professionals due to awareness of disease and symptoms. Incorrect use of medication caused harmful effects. To assess the knowledge, attitude and practice of health professionals, this survey was conducted. A cross-sectional study was carried out among health professionals of different specialities. Knowledge, attitude and practice-based questions were asked through an electronically distributed questionnaire. Data were statistically tested using the Chi-square test with SPSS. Most of the health professionals were aware with the term of self-medication; however the knowledge about related questions was not satisfactory. Almost half of the participants practiced self-medication. The prevalence of self-medication among participants was high. They need to be trained and educate about the incorrect use of self-medication.

## Background

The selection and use of medical products and medicine including traditional medicine and herbal medicines by individuals to treat recognized symptoms or illness or continued use of prescribed medicine for chronic symptoms or diseases are termed as self-medication. [1] According to WHO, with improving the socio-economic and education status of individuals in developing countries, it plays an important role in the health care system. [2 - check with author] In South East Asia self-medication has become very common due to high population density, lack of rules and regulations and inadequate medical facilities. [3] Self-medication can be useful for the individuals who do not have severe illness or do not seek medical attention, if appropriately practices e.g., treatment of headache by the students with paracetamol (over-the-counter product), but its inappropriate practice can be harmful because it increases the risk of drug abuse/misuse. [4 - check with author] It doesn't only harm the health of students but also might be a threat for their profession in future. [5] They can affect the care quality because they may advice relatives and patients to self-medication practice. [6] Previous studies have revealed that the use of self-medication has increased in health science students worldwide. The use of self-medication is reported in 75.2% medical students from the King Abdulaziz University, KSA [7] and 79.9% students practiced self-medication in the Serbia University of Belgrade. [8 - check with author] There are many reasons for the prevalence of self-medication i.e., to save money, previous drug experience, long waiting time at doctor clinics and mild health. [9] Headache is the most common indication that drives the students to practice self-medication and analgesic consumption. [10] Antibiotics, decongestant, analgesics, antipyretic, anti-acid and cough suppressants were the most commonly used drugs. [11] Students of health sciences have more knowledge about illness and medicines, so they are more prone to use analgesics and practice self-medication. So, they might be at risk of overuse of analgesics. [8 - check with author] There is a need to address this problem in nursing students, as they would be the healthcare providers in future. [12 - check with author] It is imperative to promote awareness of the hazards of self-medication to specialists. The basic objective of this survey is to assess the knowledge and attitude of participants to self-medication and their rate of practicing it.

## Material and Methods:

### Study design and tools:

A questionnaire-based cross-sectional study was conducted in Saudi Arabia to assess the knowledge, attitude and practice of self-medication among different healthcare providers i.e. Doctors, nurses, pharmacists and physiotherapists. Professionals who were willing to participate in the survey were included. Questions were written in the English language and had three sections (i) Knowledge related to self-medication (ii) attitude towards self-medication and (iii) it had questions about the practice. Clinical and demographic details were also asked.

### Data collection:

Total 440 professionals participated in this survey. After taking the consent, the questionnaire was electronically distributed and clear objective of the study was explained to them. Data was collected and compiled for further statistical analysis.

### Statistical analysis:

Data were analyzed statistically by using SPSS and significance of the relationship between speciality and different questions were measured with Chi-square test by taking p<0.05.

## Results:

### Demographic details:

A total of 440 professionals from different specialities participated in this study. Among them 113 (25.7%) were female and 317 (74.3%) were male. Their age was distributed, as 15-19 years were 16.1%; 20-24 years were 55.9% and 25 years and above were 28.0%. As concerned their speciality medical professionals were 90, professionals related to the dental field were 128, 74 from the pharmacy, 101 from the nursing and 47 were from the physiotherapy. 36.6%, 36.1% and 27.3% were from 3rd, 4th and 5th years students respectively (Table 1 - see PDF).

### Knowledge:

Most of the participants 70 from medical, 96 from dental, 65 from the pharmacy, 53 from nursing and 30 from physiotherapy were agreed that medication should be decided by doctor's suggestion while total 126 (28.6%) used traditional healer for medication. Majority of the participants 189 (43.0%) were not aware of self-medication and 33.6% knew about it. Medical professionals had more knowledge than others. All participants selected a different source of information. The most chosen source was advice from the pharmacist. 257 (58.4%) participants chose this source, 40 (9.2%) selected advice from friends and relatives, 56 (12.7%) said that people used self-medication from their own experience and 39 (8.8%) agreed that this source is reading material and medical books. Knowledge about a medical condition which leads the use of self-medication was as follow; headache/fever for 286 (65.0%), Gl problems for 57 (13.0%) and skin problems for 39 (8.8%). Most of the participants from all field (24.1% from medical, 30.8% from dental, 21.7% from the pharmacy, 15.7% from nursing and 8.7% from the pharmacy) were agreed that headache/fever is the most common reason for which people practice self-medication. The maximum number of individual who considered nausea and vomiting as a side effect was 75 (31.1%) from medical, palpitation for 11(26.8%) from physiotherapy, 15 (31.3%) from pharmacy chose dizziness as a side effect of self-medication. 229 participants out of 440 didn't have the knowledge about an over-the-counter medication. Relationship between speciality and different questions were not significant, however, awareness about the term self-medication and OTC medicines was significant (Table 2 - see PDF).

### Attitude:

Out of 440 respondents 168 (38.2%) considered self-medication useful while 104 thought it useless. 54.6% of participants thought that reason for self-medication is knowledge about disease and medicine. Previous experience, timesaving, low cost and experience of family/friends/relatives was the reason for 68 (15.5%), 49 (11.1%), 41(9.3%) and 42 (9.5%) respectively. There is no significant relationship between attitude and speciality of participants (Table 3 - see PDF).

### Practice:

**Figure 4:**
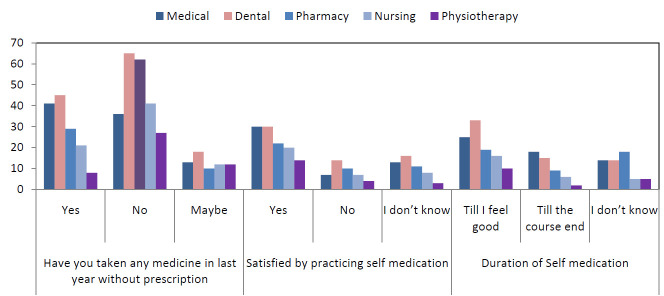
Self-medication practice of participants

Most of the participant (52.5%) used any medication in the last year by doctor's prescription only 47.5% practiced self-medication in last year without doctor's prescription. Out of this 47.5%, most commonly (27.1%) practiced medicine was Analgesic/NSAIDs followed by Vitamins and supplements (20.2%), Antipyretic (12.7%), both Antibiotic and Cough syrup (10.8%), Anti-allergic (9.7%) and Anxiolytic (8.6%) (Figure 4). 103 (23.4%) respondents said that they practice self-medication till they feel good while 11.4% practiced it till the course end. comparison of practice with speciality was non-significant (Table 4 - see PDF).

## Discussion:

Self-medication means the treatment of different illness and diseases without the suggestion of a doctor. Its inappropriate use can lead to harmful effects on health. Total of 440 health professionals from different specialities participated in this survey. They have adequate knowledge about this term, which is similar to the findings of other studies. [13,14 - check with author] It has been revealed in this study that most common reason for the use of self-medication was headache/fever, which is in line with the findings of Shah et al. [15] that concluded that most frequent condition for self-medication. Symptoms for which students tend for self-medication were cold, vomiting, hyperacidity and fever. [16] In the same study [15] it has been found that antipyretics and analgesics were the commonly used drugs. Same results were found in the current study. Other studies also proved that use of antipyretics and analgesics. [17,18 - check with author] Despite having the knowledge bout SM, a large number of participants showed a negative attitude, they considered it very useful or useful. This was similar to the study of who reported negative attitude and inappropriate practice among health science students. [19] Most of the participants 52.5% in this survey didn't practiced SM. This result can be compared with the other findings where same or higher use of self-medication was reported. [20] Particularly, this range lied between 51% and 75% in Saudi Arabia. [21] There is no significant association between speciality and self-medication practice in the current study. Similarly, in another survey, no relationship was found between high medical knowledge and self-medication. [22] The dispense of antibiotics is alarmingly high despite regulations mandated by the Saudi Authorities [23].

## Conclusion:

Individuals who participated in the survey had a reasonable amount of knowledge about self-medication. However, attitude toward SM and its practice needs to be addressed. More seminars and conferences should be arranged to highlight this issue in professionals as well as in the general public. The dispense of OTC products should be strictly regulated.

## Figures and Tables

**Figure 1:**
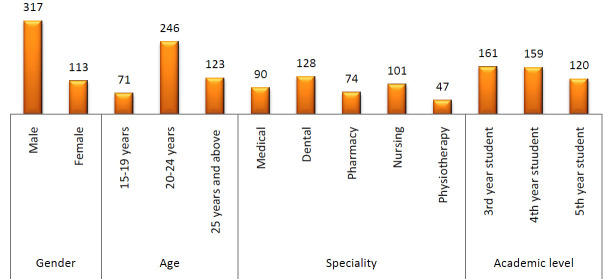
Demographic details of Participants

**Figure 2:**
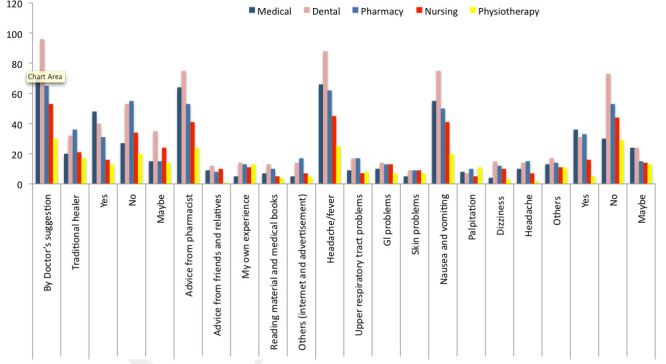
Knowledge on self-medication by medical students

**Figure 3:**
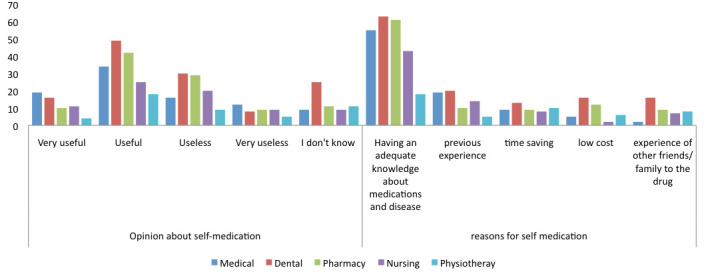
Attitude of participants towards self-medication

**Figure 5:**
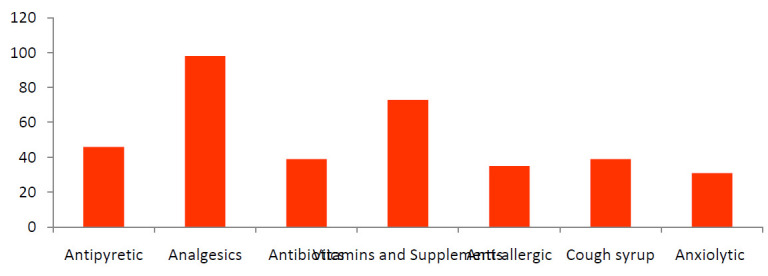
Distribution of medicines used as self-medication
